# Cardioprotective Potential of Human Endothelial-Colony Forming Cells from Diabetic and Nondiabetic Donors

**DOI:** 10.3390/cells9030588

**Published:** 2020-03-02

**Authors:** Marcus-André Deutsch, Stefan Brunner, Ulrich Grabmaier, Robert David, Ilka Ott, Bruno C. Huber

**Affiliations:** 1 Department of Thoracic and Cardiovascular Surgery, Heart and Diabetes Center NRW, Ruhr-University Bochum, Georgstr. 11, D-32545 Bad Oeynhausen, Germany; mdeutsch@hdz-nrw.de; 2 Department of Internal Medicine I, Ludwig-Maximilians-University, Campus Grosshadern, Marchioninistr. 15, D-81377 Munich, Germany; stefan.brunner@med.uni-muenchen.de (S.B.); ulrich.grabmaier@med.uni-muenchen.de (U.G.); 3 Reference- and Translation Center for Cardiac Stem Cell Therapy (RTC), Rostock University Medical Center, Department of Cardiac Surgery, Department Life, Light & Matter (LL&M), 18057 Rostock, Germany; Robert.David@med.uni-rostock.de; 4 Department of Internal Medicine, Division of Cardiology, Helios Klinikum Pforzheim, Kanzlerstraße 2-6, D-75175 Pforzheim, Germany; ilka.ott@helios-gesundheit.de

**Keywords:** cardiovascular diseases, adult stem cells, cardiac regeneration, myocardial infraction, angiogenesis

## Abstract

Objective: The potential therapeutic role of endothelial progenitor cells (EPCs) in ischemic heart disease for myocardial repair and regeneration is subject to intense investigation. The aim of the study was to investigate the proregenerative potential of human endothelial colony-forming cells (huECFCs), a very homogenous and highly proliferative endothelial progenitor cell subpopulation, in a myocardial infarction (MI) model of severe combined immunodeficiency (SCID) mice. Methods: CD34^+^ peripheral blood mononuclear cells were isolated from patient blood samples using immunomagnetic beads. For generating ECFCs, CD34^+^ cells were plated on fibronectin-coated dishes and were expanded by culture in endothelial-specific cell medium. Either huECFCs (5 × 10^5^) or control medium were injected into the peri-infarct region after surgical MI induction in SCID/beige mice. Hemodynamic function was assessed invasively by conductance micromanometry 30 days post-MI. Hearts of sacrificed animals were analyzed by immunohistochemistry to assess cell fate, infarct size, and neovascularization (huECFCs *n* = 15 vs. control *n* = 10). Flow-cytometric analysis of enzymatically digested whole heart tissue was used to analyze different subsets of migrated CD34^+^/CD45^+^ peripheral mononuclear cells as well as CD34^−^/CD45^−^ cardiac-resident stem cells two days post-MI (huECFCs *n* = 10 vs. control *n* = 6). Results: Transplantation of human ECFCs after MI improved left ventricular (LV) function at day 30 post-MI (LVEF: 30.43 ± 1.20% vs. 22.61 ± 1.73%, *p* < 0.001; ΔP/ΔT_max_ 5202.28 ± 316.68 mmHg/s vs. 3896.24 ± 534.95 mmHg/s, *p* < 0.05) when compared to controls. In addition, a significantly reduced infarct size (50.3 ± 4.5% vs. 66.1 ± 4.3%, *p* < 0.05) was seen in huECFC treated animals compared to controls. Immunohistochemistry failed to show integration and survival of transplanted cells. However, anti-CD31 immunohistochemistry demonstrated an increased vascular density within the infarct border zone (8.6 ± 0.4 CD31^+^ capillaries per HPF vs. 6.2 ± 0.5 CD31^+^ capillaries per HPF, *p* < 0.001). Flow cytometry at day two post-MI showed a trend towards increased myocardial homing of CD45^+^/CD34^+^ mononuclear cells (1.1 ± 0.3% vs. 0.7 ± 0.1%, *p* = 0.2). Interestingly, we detected a significant increase in the population of CD34^−^/CD45^−^/Sca1^+^ cardiac resident stem cells (11.7 ± 1.7% vs. 4.7 ± 1.7%, *p* < 0.01). In a subgroup analysis no significant differences were seen in the cardioprotective effects of huECFCs derived from diabetic or nondiabetic patients. Conclusions: In a murine model of myocardial infarction in SCID mice, transplantation of huECFCs ameliorated myocardial function by attenuation of adverse post-MI remodeling, presumably through paracrine effects. Cardiac repair is enhanced by increasing myocardial neovascularization and the pool of Sca1^+^ cardiac resident stem cells. The use of huECFCs for treating ischemic heart disease warrants further investigation.

## 1. Background

Ischemic heart disease following acute myocardial infarction (AMI) is the leading cause of morbidity and mortality in the Western world [1]. Most of the clinically approved therapeutics focus on modulating hemodynamics to reduce early mortality but do not facilitate cardiac repair, which would be needed to reduce the incidence of heart failure [2]. Therefore, the concept of cell-based therapies may have the potential to transform the treatment and prognosis of heart failure through regeneration or repair of injured cardiac tissue [3,4]. Most clinical trials have used bone marrow derived mononuclear cells (BMCd), which have demonstrated inconsistent and, overall, modest efficacy [5].

In recent years, there has been an intense investigative effort to uncover the mechanism by which transplanted stem cells preserve the function of infarcted hearts. Based on these findings, the attenuation of ischemic cardiomyopathy after cell transplantation is not attributable to cardiomyocyte repopulation or transdifferentiation. Rather, functional benefits after stem cell transplantation might be attributable to an augmentation of the natural process of myocardial healing by paracrine signaling and promoting neovascularization [6].

Endothelial progenitor cells (EPCs) are a minor population of mononuclear cells migrating from the bone marrow into the bloodstream, which are able to home in on sites of injury and promote neovascularization, which finally is associated with increased blood flow and tissue repair [7]. EPCs can be isolated from peripheral or umbilical cord blood and culturing in vitro can produce two different EPC types. These two distinct EPC subtypes have been named as early EPCs (eEPCs) and late outgrowth EPCs (LOEPCs) or endothelial colony-forming cells (ECFCs). ECFCs comply with the definition of bona fide EPCs and represent a well-characterized and homogeneous cell population of endothelial origin with high proliferative capacity and inherent vasculogenic activity [8,9,10]. In vivo, their potential has been investigated in different mouse models where ECFCs increased neovascularization and tissue regeneration [11]. In a rat stroke model, ECFCs treatment was associated with reduced glial scarring and increased functional recovery, which could be explained by stimulation of angiogenesis and a marked reduction in apoptosis [12]. However, there are only a few studies investigating the potential of human ECFCs to regenerate ischemic myocardium. To create a clinically relevant scenario, which aims to address the effect of cell therapy after acute MI, we transplanted ECFCs from patients with coronary artery disease (CAD) into immunodeficient SCID mice and employed functional studies, immunohistology, as well as flow cytometry to assess their potential in facilitating cardiac regeneration.

## 2. Methods

### 2.1. Isolation and Culture of ECFCs

Human adult ECFCs were collected by leukapheresis after G-CSF-induced mobilization of CD34^+^ cells from diabetic (*n* = 9) and nondiabetic (*n* = 8) patients with coronary artery disease. The diagnosis of diabetes was made in accordance with current guidelines (mean HbA1c 7.5% ± 0.3%). 

For ECFC collection, mononuclear cells from leukapheresis were isolated by density gradient centrifugation for 20 min at 1000× *g* (Ficoll-Hypaque, Seromed, Berlin, Germany). CD34^+^ cells were isolated using immunomagnetic beads (Miltenyi Biotec, Bergisch Gladbach, Germany) [13]. The purity of the isolated CD34^+^ cells ranged between 86% and 99% as assessed by flow cytometry (EPICS XL, Couter, Hialeah, FL, USA). This study was approved by the Medical Ethics Committee of the Technical University of Munich.

CD34^+^ cord blood (CB) and peripheral blood (PB) cells were cultured using a modified protocol as described in [14]. Briefly, CD34^+^ cells from mobilized PB was cultured on 1% gelatin (Sigma, Hamburg, Germany) or fibronectin (10 μg/cm^2^, Cellsystems, St. Katharinen, Germany) in Iscove’s Modified Dulbecco’s Medium (IMDM, Gibco, Paisley, UK), with 10% horse serum and 10% fetal calf serum (PAN-Biotech, Aidenbach, Germany) supplemented with penicillin/streptomycin (Gibco), 50 ng/mL recombinant human stem cell factor (SCF, R&D Systems, Abingdon, UK), 50 ng/mL vascular endothelial growth factor (VEGF, R&D Systems), 20 ng/mL basic fibroblast growth factor (FGF-2, R&D Systems), and 20 ng/mL stem cell growth factor (SCGFβ, Peprotech, London, UK). This medium (ECM) was replaced 3 times a week. After 3 weeks, cells were adapted from ECM to the low-serum EGM-2 medium (Cellsystems). To analyze EC colony-forming units (CFU-EC), CD34^+^ cells were plated in a limiting dilution series of cell concentrations in 24-well plates and treated as above. These multiwell tissue culture plates were scored for the presence (“positive”) or absence (“negative”) of EC colonies between 21 and 35 days. Adherent cells were cultured to confluence in 1% gelatin-coated chamber slides (Nalge Nunc, Naperville, IL, USA). Cells were washed twice in phosphate-buffered saline (PBS), fixed, and permeabilized using Fix and Perm (Dianova, Hamburg, Germany). Samples were then incubated for 2 h with primary antibodies: antihuman specific CD31 (Sertotec, Raleigh, NC, USA), anti-CD105, anti-CD144 (VE-cadherin, Coulter-Immunotech, Krefeld, Germany), anti-CD45 and anti-vWF (Dako, Hamburg, Germany), anti VEGF-R2 (KDR-1 and KDR-2, Sigma), anti-Flt1, anti-Flt4, anti-Tie-2 (Santa Cruz Biotechnologies Inc., Heidelberg, Germany), and CD146 (Chemicon, Limburg, Germany). To visualize antibody binding (mouse and rabbit antibodies), the peroxidase-labeled avidin-biotin method (Universal Dako LSAB^®^-Kit, Dako, Santa Clara, CA, USA) was used according to the manufacturer’s recommendations. For goat primary antibodies, donkey antigoat antibodies directly conjugated to peroxidase were used (Jackson Laboratories, Dianova, Hamburg, Germany). Isotype-matched control antibodies (Coulter-Immunotech, Jackson Laboratories, Brea, CA, USA) served as negative controls. In selected experiments, nuclear staining was performed with hematoxylin staining solution (Merck, Darmstadt, Germany).

### 2.2. Animal Model

For this study, we used 8–12 weeks old male severe combined immunodeficiency (SCID)beige mice (Charles River, Wilmington, MA, USA), which were kept under pathogen-free conditions. MI was induced by surgical occlusion of the left anterior descending artery (LAD) through a left anterolateral approach as described previously [15,16]. Briefly, animals were anesthetized with a mixture of 100 mg/kg ketamine (Sigma, St. Louis, MO, USA) and 5 mg/kg xylazine (Sigma) intraperitoneally. Subsequently, they were intubated and artificially ventilated with room air at 200 breaths/min using a mouse ventilator (HUGO SACHS, March-Hugstetten, Germany). A left anterolateral thoracotomy was performed, and MI was induced by surgical occlusion of the left anterior descending artery (LAD) with an 8-0 Prolene suture. Animal care and all experimental procedures were performed in strict accordance with the German and National Institutes of Health animal legislation guidelines and were approved by the local animal care committees (AZ 209.1/211-2531-117/02). The investigation conforms to the Guide for the Care and Use of Laboratory Animals published by the US National Institutes of Health (NIH Publication No. 85-23, revised 1996). 

### 2.3. Cell Delivery

For cell delivery, a 15 µL suspension containing 5 × 10^5^ human ECFCs or a 15 µL saline solution was administered directly after LAD ligation by two injections in the border zone of the infarcted myocardium using a 10 µL 32G Hamilton syringe (Reno, NV, USA). One injection was performed on the medial and one at the lateral side of the infarcted area.

### 2.4. Invasive Evaluation of Cardiac Function

For evaluation of myocardial function, mice of the previously described groups underwent impedance-micromanometer catheterization. The method as well as data analyses were performed as previously described in the literature [17,18]. Briefly, the animals were anesthetized with thiopental (100 mg/kg intraperitoneal) and ventilated using a mouse ventilator (HUGO SACHS). After that, a 1.4 French impedance-micromanometer catheter (Millar Instruments, Houston, TX, USA) was introduced into the left ventricle retrogradely via the right carotid artery, and pressure—volume loops were recorded. The method was based on measuring the time-varying electrical conductance signal of two segments of blood in the left ventricle from which total volume is calculated. Raw conductance volumes were corrected for parallel conductance by the hypertonic saline dilution method [15].

### 2.5. Flow Cytometry of Nonmyocyte Cardiac Cells

We previously hypothesized that EPC may not be responsible only for the formation of new vessels but may also recruit local cells [13]. Therefore, we performed flow cytometry in order to evaluate the effects of cell transplantation on proangiogenic cardiac cell populations.

Hearts of the mice were investigated by flow cytometry (FACS) as described previously [19]. Briefly, for cardiac FACS analyses, infarcted hearts of the mice were explanted at day 2 and retrogradely perfused with saline (0.9% NaCl) to wash out circulating blood cells. Thereafter, a “myocyte-depleted” cardiac cell suspension was prepared, incubating minced myocardium in 0.1% collagenase IV (Gibco, Co Dublin, Ireland) for 30 min at 37 °C, lethal to most adult mouse cardiomyocytes. Cells from peripheral blood and hearts were incubated for 40 min in the dark at 4 °C with the following fluoresceinisothiocyanate (FITC), phycoerythrin (PE), and peridininchlorophyll-protein (PerCP) conjugated monoclonal antibodies: CD45-PerCP, CD34-FITC, and CXCR4-PE (all from BD Pharmingen). A matching isotype antibody served as control. Cells were analyzed by 3-color flow cytometry using a Coulter^®^ Epics^®^ XL-MCLTM flow cytometer (Beckman Coulter, Brea, USA). Each analysis included 50,000 events.

### 2.6. Histology and Immunohistochemical Analyses

Infarct size was calculated as the average of four coronal sections sampled at 2 mm intervals from the apex to the base using the following Equation (1) developed by Pfeffer et al. [20]:

(1) $$Infarct\;Size=\frac{Coronal\;Infarct\;Perimeter\;\left(Epicardal+Endocardial\right)\;}{Total\;Coronal\;Perimeter\;\left(Epicardal+Endocardial\right)}\times 100$$

infarct size ¼ [coronal infarct perimeter (epicardial plus endocardial)/total coronal perimeter (epicardial plus endocardial)] × 100. Infarct wall thickness was measured in Masson’s trichrome stained sections by taking the average length of five segments along evenly spaced radii from the centre of the LV through the infarcted free LV wall [15]. To assess the incorporation and phenotype of injected EPCs in infarcted myocardium, we performed standard histological procedures (hematoxylin/eosin and Masson Trichrome) and immunostaining, which was performed as follows:

For immunohistochemical analyses, hearts were fixed in 4% phosphate-buffered formalin overnight and embedded in paraffin as described previously [15]. Before immunostaining, mounted tissue sections were deparaffinized by rinsing 3× for 5 min in Xylene followed by 2× for 5 min 100%, 2× for 5 min 96%, and 2× for 5 min 70% ethanol rinses. Endogenous peroxidases were quenched in 7.5% H_2_O_2_ in distilled water for 10 min. Following that, slides were rinsed in distilled water for 10 min and twice in TRISbuffer (pH 7.5) for 5 min. Finally, sections were incubated at room temperature for 60 min with either a primary antibody detecting vimentin (monoclonal mouse anti-human; Dako, Glostrup, Denmark) or class I human leukocyte antigen (HLA) (monoclonal mouse anti-human HLA-A, B, C; WAK-Chemie, Steinbach, Germany). Pretreatment was performed for 30 min (microwave 750 W) using citrate buffer (10 mM, pH 6.0) for vimentin or a target retrieval solution (Dako) for HLA-A,B,C, respectively. The detection system for vimentin and HLA-A, B, C was Dako REAL and APAAP mouse.

### 2.7. Statistical Analyses

Results were expressed as mean ± SEM. Multiple group comparisons were performed by one-way analyses of variance (ANOVA) followed by the Bonferroni procedure for comparison of means. Comparisons between two groups were performed using the unpaired Student’s *t*-test. Data were considered statistically significant at a value of *p* ≤ 0.05.

## 3. Results

### 3.1. Improvement of Cardiac Function after Intramyocardial Injection of Human ECFCS into Ischemic Myocardium

**Figure 1:**
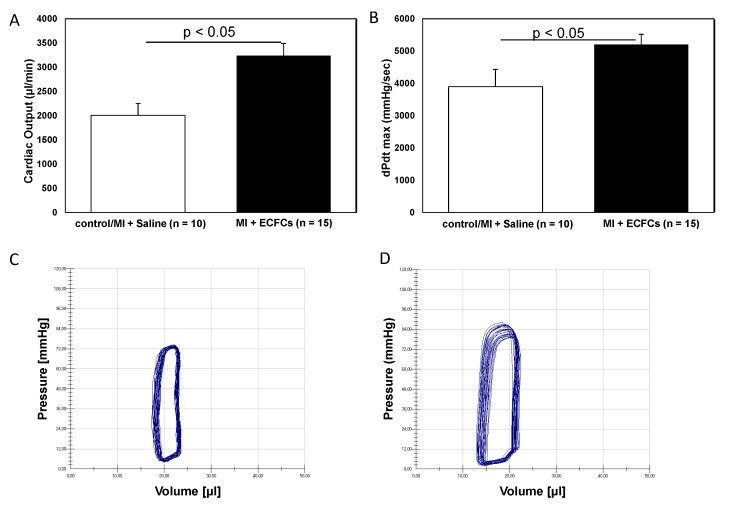

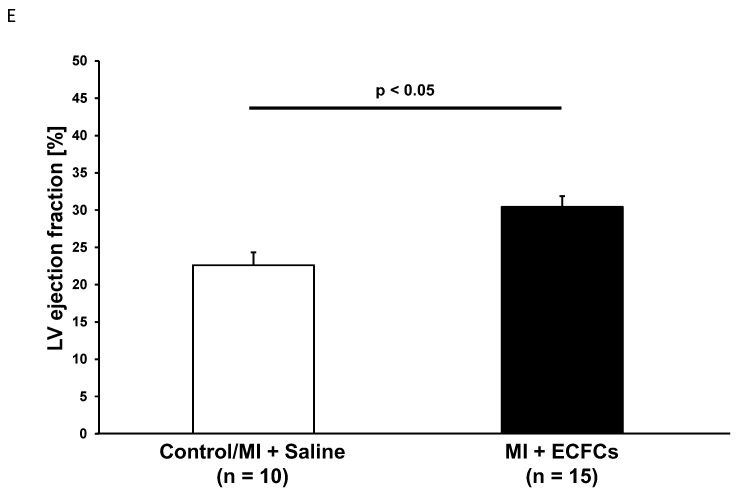
Improved myocardial function after human endothelial colony-forming cells (ECFC) transplantation into ischemic myocardium. Bar graphs representing cardiac output (**A**), contractility (**B**), and ejection fraction (**E**) in saline-treated animals (white bars) and ECFC-treated animals (black bars) 30 days after myocardial infarction (MI). Data represent mean ± SEM; n.s., not significant. Representative pressure volume loops of saline-treated animals (**C**) and ECFC-treated animals (**D**) 30 days after MI.

To investigate the regenerative potential of human ECFCs transplanted intramyocardially, 5 × 10^5^ cells were injected directly after surgical occlusion of the LAD and functional parameters, and histological and immunohistochemical data were collected. To assess the functional parameters, pressure-volume relations of ECFC-treated and saline-treated hearts were measured at day 30 after MI using conductance catheters (Figure 1). Heart rates did not differ significantly between the groups, showing that experimental conditions such as anesthesia did not influence the measurements (data not shown). At day 30 after MI, we detected a significant improvement in cardiac contractility (Figure 1A; dPdt max: 5202.28 ± 316.68 mmHg/s vs. 3896.24 ± 534.95 mmHg/s, *p* = 0.03) in human ECFC-treated mice compared to saline-treated animals. Moreover, compared to saline-treated control animals, human ECFC-treated mice revealed significantly improved stroke work 30 days after MI (CO 3470.70 ± 254.44 µL/min vs. 2006.71 ± 243.18 µL/min; *p* < 0.001, Figure 1B–D). Accordingly, ECFC treatment was associated with improved LV ejection fraction (Figure 1E, LVEF 30.43 ± 1.20% vs. 22.61 ± 1.73%; *p* < 0.001).

### 3.2. Attenuated Infarct Remodeling after Transplantation of Human ECFCS into Ischemic Myocardium

**Figure 2:**
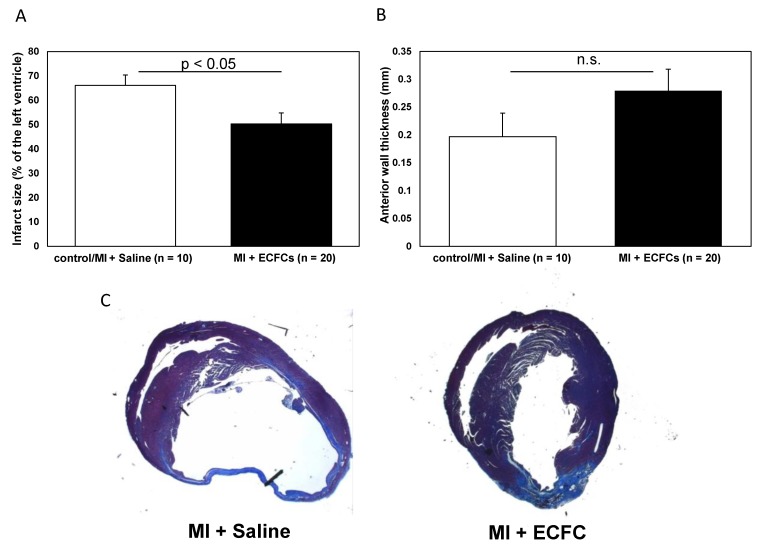
Attenuated infarct remodeling after human ECFC transplantation into ischemic myocardium. (**A**) Bar graphs representing the size of infarction (%) in saline-treated animals (white bar) and ECFC-treated animals (black bar) 30 days after MI. (**B**) Bar graphs representing anterior wall thickness (mm) in saline-treated animals (white bar) and ECFC-treated animals (black bar) 30 days after MI. Data represent mean ± SEM; n.s., not significant. (**C**) Representative Masson trichrome staining of infarcted hearts 30 days after MI.

After functional profiling, we performed histological analysis of explanted hearts at day 30 following MI and cell transplantation. As reported in prior studies [15,19], permanent occlusion of the LAD artery resulted in a typical pattern of injury with transmural involvement of the myocardium in regions supplied by the main branches of the left coronary artery. Histological analyses revealed less pronounced thinning of the LV anterior wall after treatment with ECFCs (0.28 ± 0.08 mm vs. 0.20 ± 0.04 mm; not significant). However, this difference did not reach statistical significance. Infarction size was significantly diminished among human ECFC-treated animals compared to control animals 30 days after MI (50.3 ± 4.5% vs. 66.1 ± 4.3%, *p* < 0.05, Figure 2). Thirty days post-MI, as assessed by human-specific antibodies against HLA and vimentin, we were not able to detect any retained ECFCs (data not shown).

### 3.3. Increased Neovascularization after Intramyocardial Injection of Human ECFCS into Ischemic Myocardium 

**Figure 3:**
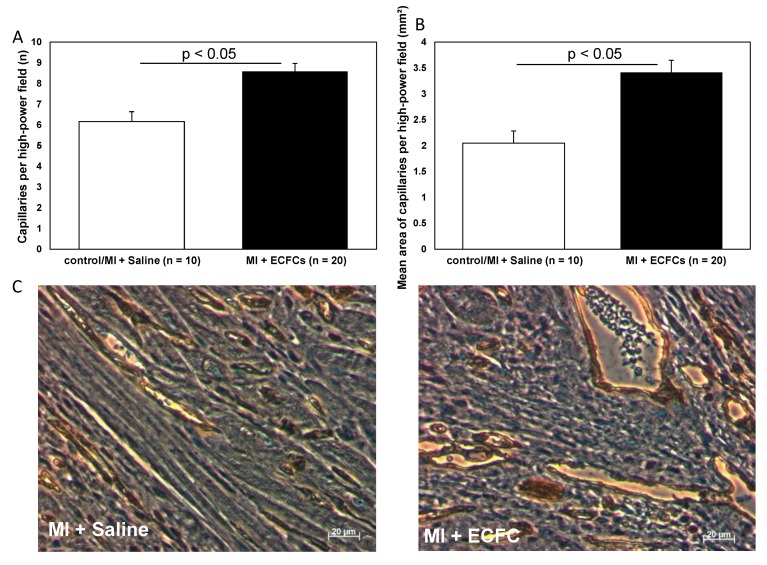
Increased neovascularization after transplantation of human ECFCs into ischemic myocardium. Histograms showing the numbers (**A**) and mean area (**B**) of CD31^+^ capillaries at the infarct border zone of saline-treated control animals (white bars) and after ECFC transplantation (black bars) 30 days after MI. Data represent mean ± SEM. (**C**) Representative immunohistochemical staining of CD31 (brown) in infarcted hearts 30 days after MI.

We hypothesized that attenuated cardiac remodeling after transplantation of ECFCs might be a result of cell-induced enhanced neovascularization. Therefore, we performed anti-CD31 immunohistochemistry to analyze the extent of neovascularization in the border zone of animals treated with ECFCs and control animals. Consistent with the smaller infarct size after ECFC therapy, heart sections of these animals revealed a significantly increased capillary density at the infarct border zone compared to the control animals (8.6 ± 0.4 vs. 6.2 ± 0.5, *p* < 0.001, Figure 3) 30 days post-MI. In accordance to these data on enhanced neovascularization after cell transplantation in vivo, we detected increased expression of the proangiogenic transcription factors HIF-1alpha and MMP-2 in ECFCs compared to human umbilical vein endothelial cells (HUVEC) cells assessed by qPCR in vitro (Figure S1A,B).

### 3.4. No Enhanced Homing of BM-Derived Progenitor Cells after Human ECFC Injection into Ischemic Myocardium

**Figure 4:**
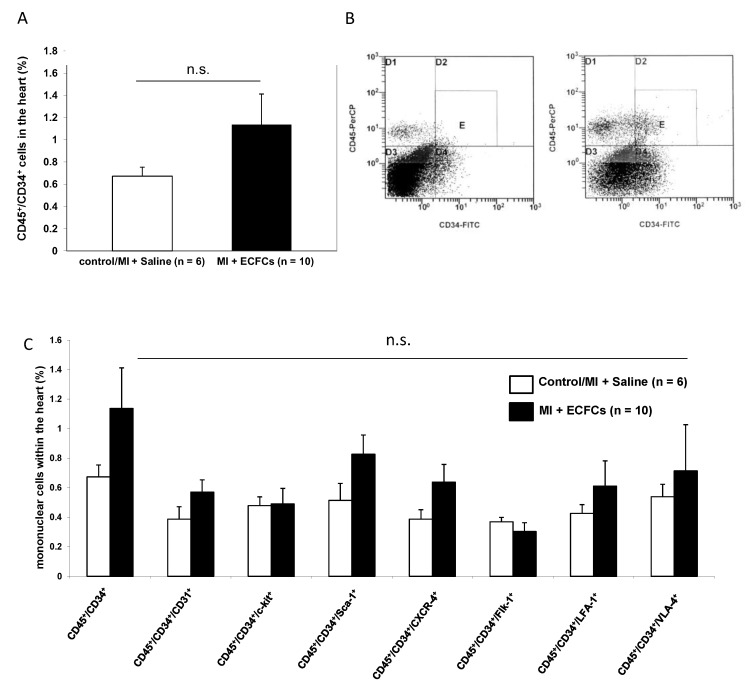
No enhanced homing of bone marrow (BM)-derived progenitor cells after human ECFC injection into ischemic myocardium. (**A**) Bar graphs representing the percentage of CD45^+^/CD34^+^ stem cells in the ischemic hearts of control animals (white bar) and after ECFC transplantation (black bar) 2 days after MI. Data represent mean ± SEM; n.s., not significant. (**B**) Representative flow cytometry (FACS) analyses of CD45^+^/CD34^+^ cells in the heart of control animals (left) and after ECFC transplantation (right) 2 days after MI. (**C**) Bar graphs representing the percentage of CD45^+^/CD34^+^ subpopulations in the ischemic hearts of control animals (white bars) and after ECFC transplantation (black bars) 2 days after MI. Data represent mean ± SEM;n.s., not significant.

Because circulating BMCs cells are known carriers of angiogenic growth factors, we sought to address whether transplantation of ECFCs is able to attract BMCs from the peripheral blood to the ischemic myocardium, thereby facilitating augmented neovascularization. To address this question, we isolated a myocyte-depleted fraction of cardiac cells and performed flow cytometry respectively. In a first step, we were interested in the amount of CD45^+^/CD34^+^ BMCs within the ischemic myocardium. Transplantation of ECFCs was associated with increased number of cardiac homing of CD45^+^/CD34^+^ cells (1.1 ± 0.3% vs. 0.7 ± 0.1%), but the values did not reach statistical significance (Figure 4A,B). Next, we further characterized CD45^+^/CD34^+^ cells utilizing the additional markers CD31, c-kit, Sca-1, CXCR4, Flk-1, LFA-1, and VLA-4. Compared to PBS treated controls, transplantation of ECFCs increased the number of all subfractions without reaching statistical significance (Figure 4C). Interestingly, among the different subfractions, the CD45^+^/CD34^+^/Sca-1 and CD45^+^/CD34^+^/CXCR4^+^ showed the highest enrichment (CD45^+^/CD34^+^/Sca-1 + 0.8 ± 0.1% vs. 0.5 ± 0.1% and CD45^+^/CD34^+^/CXCR4^+^ 0.6 ± 0.1% vs. 0.4 ± 0.1%). 

### 3.5. Increased Numbers of Sca-1 Positive Resident Cardiac Stem Cells after ECFCS Injection into Ischemic Myocardium

**Figure 5:**
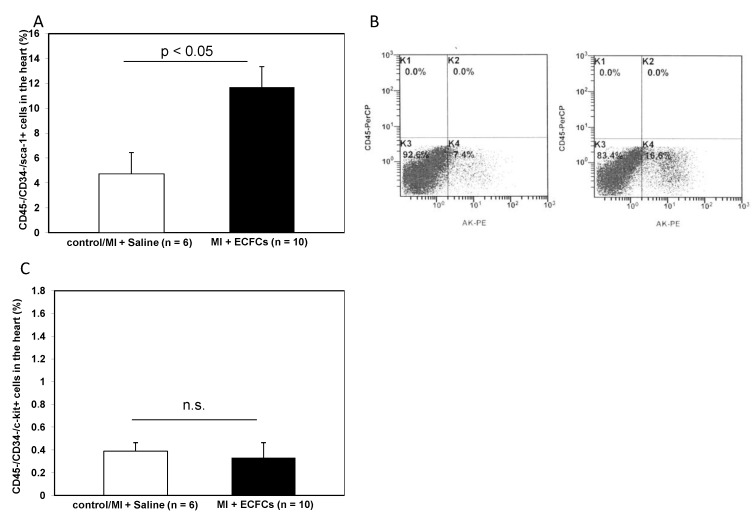
Increased numbers of Sca-1^+^ resident cardiac stem cells after ECFC injection into ischemic myocardium. (**A**) Bar graphs representing the percentage of Sca-1^+^ resident cardiac stem cells in the ischemic hearts of control animals (white bar) and after ECFC transplantation (black bar) 2 days after MI. Data represent mean ± SEM. (**B**) Representative FACS analyses of Sca-1^+^ resident cardiac stem cells in the heart of control animals (left) and after ECFC transplantation (right) 2 days after MI. (**C**) Bar graphs representing the percentage of c-kit^+^ resident cardiac stem cells in the ischemic hearts of control animals (white bar) and after ECFC transplantation (black bar) 2 days after MI. Data represent mean ± SEM; n.s., not significant.

In recent years, there has been emerging evidence that the heart contains a reservoir of resident cardiac progenitor cells [21]. These cells are positive for various markers, such as c-kit or Sca-1. We hypothesized that these resident cells may play a role in the repair of the injured heart, i.e., by secretion of angiogenic growth factors and contribution to improved neovascularization. To investigate these cells, we analyzed the fraction of CD45^−^/CD34^−^ cells within the ischemic myocardium of control and ECFC-treated animals and further characterized cells that additionally expressed c-kit or Sca-1. Transplantation of ECFCs significantly increased the number of CD45^−^/CD34^−^/Sca-1^+^ progenitor cells 2 days after myocardial ischemia compared to controls (11.70 ± 1.67% vs. 4.47 ± 1.71%, *p* < 0.05). In contrast, no difference in the number of CD45^−^/CD34^−^/c-kit^+^ could be observed in ECFC-treated compared to control animals (0.33 ± 0.11% vs. 0.40 ± 0.06%; not significant). Results are depicted in Figure 5. 

### 3.6. No Difference in Therapeutic Effectiveness of ECFCS Derived from Diabetic or Nondiabetic Donors after Transplantation into Ischemic Myocardium

**Figure 6:**
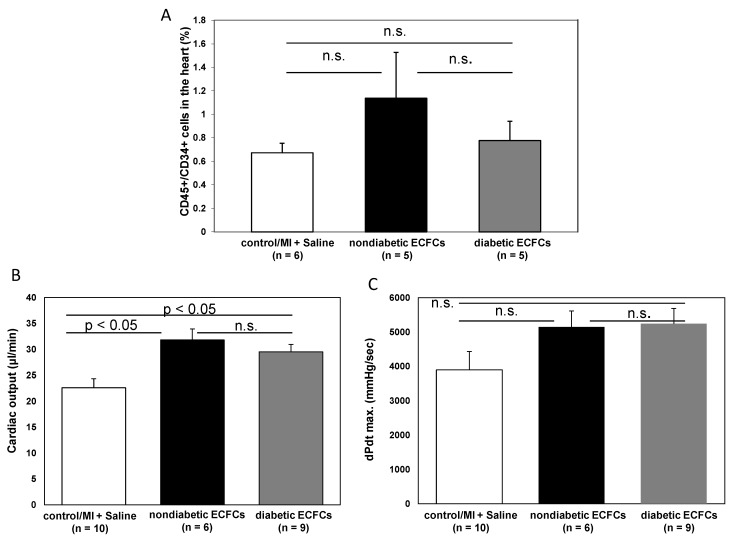

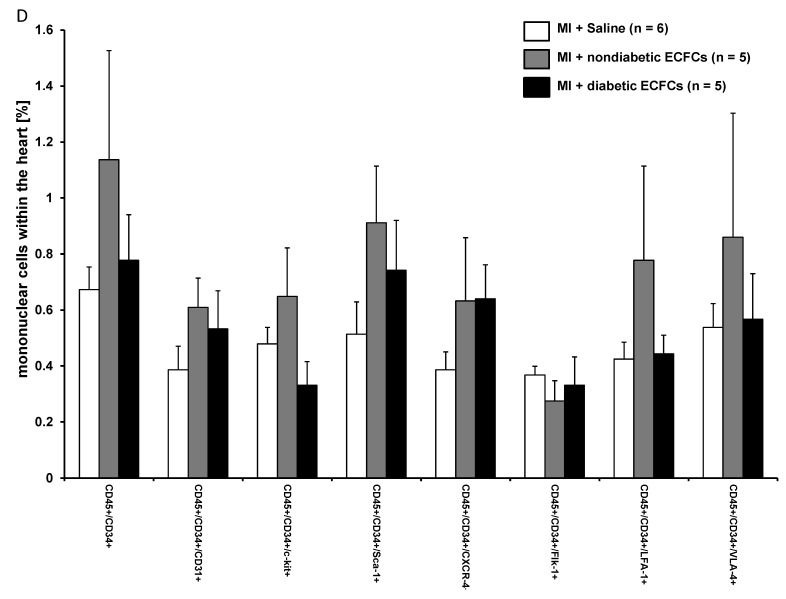
No difference in therapeutic effectiveness of ECFCs derived from diabetic or nondiabetic donors after transplantation into ischemic myocardium. (**A**) Bar graphs representing the percentage of CD45^+^/CD34^+^ stem cells in the ischemic hearts of control animals (white bar) after ECFC transplantation from nondiabetic donors (black bar) and after ECFC transplantation from diabetic donors (grey bar) 2 days after MI. Data represent mean ± SEM; n.s., not significant. (**B**) Bar graphs representing cardiac output and contractility (**C**) of control animals (white bar) after ECFC transplantation from nondiabetic donors (black bars) and after ECFC transplantation from diabetic donors (grey bars) 30 days after MI. Data represent mean ± SEM; n.s., not significant. (**D**) Bar graphs representing the percentage of CD45^+^/CD34^+^ subpopulations in the ischemic hearts of control animals (white bar) after ECFC transplantation from nondiabetic donors (black bars) and after ECFC transplantation from diabetic donors (grey bars) 2 days after MI. Data represent mean ± SEM; n.s., not significant.

To evaluate the hypothesis that diabetes mellitus may impair the protective potential of ECFCs, we stratified cell donor patients into “diabetic” versus “nondiabetic”. Interestingly, there was no statistical difference in the amount of CD45^+^/CD34^+^ BMCs within the ischemic myocardium between animals treated with either ECFCs from diabetic or nondiabetic patients (1.1 ± 0.4 (non DM-ECFCs) vs. 0.8 ± 0.2 (DM-ECFCs) vs. 0.7 ± 0.1% (control); for non DM-ECFCs vs. DM-ECFCs *p* = 0.18). Furthermore, LV function assessed by catheterization revealed no significant difference between animals transplanted with ECFC from diabetic or nondiabetic patients after MI. However, both ECFCs from diabetic or nondiabetic patients were able to significantly improve cardiac contractility compared to saline-treated control animals (dPdt max: 5154.65 ± 469.37 (non DM-ECFCs) vs. 5240.02 ± 447.51 (DM-ECFCs) vs. 3896.24 ± 534.95 (control) mmHg/s, for nonDM-ECFCs vs. DM-ECFCs *p* = 0.28; CO: 3821.92 ± 238.14 (non DM-ECFCs) vs. 2006.71 ± 243.18 vs. 3236.54 ± 384.78 (DM-ECFCs); for nonDM-ECFCs vs. DM-ECFCs *p* = 0.28; Figure 6).

## 4. Discussion

This study aimed to evaluate a proregenerative/reparative potential of human ECFCs in a murine model of MI. Our main findings were the following: (1) transplantation of human ECFCs into ischemic myocardium resulted in an improved cardiac function and reduced size of infarction 30 days after MI; (2) the attenuated remodeling after ECFC transplantation was associated with enhanced neoangiogenesis; (3) treatment with ECFCs increased the number of resident Sca-1^+^ stem cells without significant homing of migrated BMCs; and (4) there was no difference in therapeutic effectiveness between transplanted ECFCs derived from diabetic or nondiabetic donors. 

In the present study, we used immunocompromised SCID animals to prevent cell death due to cell rejection. However, 30 days after MI, we were not able to detect any transplanted cells by histological analysis. Sheikh et al. [6] investigated recently the survival kinetics and gene expression changes of transplanted BMMCs after transplantation into ischemic myocardium. Utilizing molecular-genetic bioluminescence imaging, they demonstrated short-lived survival of cells following transplant, with less than 1% of cells surviving by six weeks post-transplantation [6]. In line with these data, Higuchi et al. [22] showed in a rat study of chronic myocardial ischemia that human EPCs transduced with the sodium iodide symporter (NIS) gene for reporter gene imaging by (124)I-PET and labeled with iron oxides for visualization by MRI showed poor cell engraftment after injection. The (124)I uptake decreased on day three and was undetectable on day seven, which was confirmed by histological analysis with CD31 and CD68 antibodies [22].

However, in accordance with previous preclinical [23,24] and clinical studies, we were able to see functional improvement after transplantation of ECFCs into ischemic myocardium. These functional effects were associated with an attenuation of adverse post-MI cardiac remodeling reflected by smaller size of infarction and less pronounced thinning of the LV wall after treatment with ECFCs. Moreover, our data showed an improved neovascularization reflected by a high number of CD31^+^ vessels at the infarct border zone. It is an ongoing matter of debate how transplanted stem and progenitor cells are able to increase neovascularization. On the one hand, it has been shown that EPCs are carriers of proangiogenic cytokines and growth factors like VEGF, HGF, angiopoietins, and IGF-1 [25,26,27]. In this regard, we detected increased expression of proangiogenic transcription factors HIF-1alpha and MMP-2 in ECFCs in vitro. Dubois et al. [28] investigated the paracrine activation after ECFC transplantation in a pig model of acute MI. After infusion of autologous ECFCs, they observed decreased infarct size and a greater vascular density, which was associated with higher levels of the proangiogenic placental growth factor (PLGF) [28]. Likewise, in a rat model of transient middle cerebral artery occlusion (MCAO), injection of ECFCs was associated with a reduction in the number of apoptotic cells and an increase in capillary density at the site of injury. These effects were associated with an increased expression of growth factors VEGF and IGF-1 in the infarct area 14 days after MCAO [12]. Furthermore, neovascularization also increased cell survival, and decreased apoptosis is an important contributor of cardio-protective paracrine effects after cell transplantation. In this regard, Kim et al. [29] showed a significant decrease of terminal deoxynucleotidyl transferase dUTP nick end labeling (TUNEL)- positive nuclei after transplantation of ECFCs compared to control animals in tissue sections collected from the peri-infarct zones at day seven [29]. 

On the other hand, there is some evidence that migration and homing of BMCs as well as expansion of resident cardiac stem cells beneficially influences cardiac remodeling after MI [3]. We used cardiac FACS analysis to quantify the number of migrated CD45^+^/CD34^+^ BMCs after ECFC transplantation. We were not able to see a significant increase in the absolute number of BMCs and their subpopulations. However, we found a higher number of CD45^−^/CD34^−^/Sca-1^+^ cardiac-resident cells within the myocardium of human ECFC treated animals injected with ECFCs. In the group of mice treated with huECFC, we observed a more than 2-fold increase in the number of CD34^−^/CD45^−^/Sca1^+^ cells 2 days after MI when compared to control animals. Sca1^+^ cells within the mouse heart have been described as a multipotent stem cell population that are able to differentiate into cardiomyocytes, endothelial cells, and smooth muscle cells in vitro and after cardiac grafting. In fact, more recently and in contrast to previous reports, it has been shown that adult Sca-1^+^ cardiac-resident cells appear to lack significant cardiomyogenic potential [30]. Lineage tracing of Sca-1 expressing cells in the mouse heart revealed only a very limited ability to significantly contribute new cardiomyocytes to the heart after MI in the mouse. In fact, they demonstrated a mostly endothelial expression pattern in Sca-1^mCm^R26^dTomato^ hearts that colocalized with CD31 expression [31]. Tang et al. [32] by targeting endogenous Sca1^+^ cells in Sca1-2A-CreER; R26-tdTomato mice through pulse labeling experiments showed that 94.25 ± 0.54% tdTomato^+^ cells were CD31^+^ endothelial cells, indicating that most Sca1^+^ cells adopt an endothelial cell fate during cardiac homeostasis. By immunostaining for tdTomato and VE-cad on post-MI murine heart sections, they detected a substantial number of tdTomato^+^ coronary endothelial cells in the injured region. Roughly, two thirds of VE-cad^+^ cells expressed tdTomato, whereas 91.77 ± 1.12% tdTomato^+^ cells expressed VE-cadherin in the injured myocardium. They concluded that Sca1^+^ cardiac progenitor cells mainly differentiate into cardiac endothelial cells and fibroblasts but not cardiomyocytes during cardiac homeostasis and after injuries. In a different experimental approach, Vagnozzi et al. [33] generated Ly6a gene-targeted mice containing either a constitutive or an inducible Cre recombinase to perform genetic lineage tracing of Sca-1^+^ cells in vivo. Similarly, they observed that the contribution of endogenous Sca-1^+^ cells to the cardiomyocyte population in the heart was < 0.005% throughout all of cardiac development, with aging, or after MI. Moreover, pulse labeling of Sca-1^+^ cells with an inducible Ly6a-MerCreMer allele revealed that Sca-1^+^ cells rather represent a subset of vascular endothelial cells that expand postnatally with enhanced responsiveness to pathological stress in vivo [33]. In our study, huECFCs treatment were a potent stimulus for Sca-1^+^ endogenous angiogenic cells. Anti-CD31 immunohistochemistry confirmed improved neovascularization within the infarct border zone, thereby contributing to protection of nonischemic areas. Sca1^+^ cells also have been described as differentiating into fibroblasts that may positively act during the early phase of infarct healing, thereby mediating positive effects on post-MI adverse remodeling. 

In our study, we did not use specific cardiac stromal cell markers to define the phenotype of CD45^−^/CD34^−^/Sca-1^+^ cells found in the myocardium (along with absence of CD31 expression). It must be the goal of future studies to further characterize our population of Sca-1^+^ cells and to completely rule out that they are not vessel-related cells or part of the mobilized pool from a noncardiac origin. 

After we had established the cardioprotective potential of ECFCs, we further evaluated our ECFC patient collective for cardiovascular risk factors and discovered that a fraction of these patients had diabetes mellitus Typ II (DM Typ II) according to current guidelines. Interestingly, we were not able to detect functional or cellular differences within the ischemic myocardium between animals treated with either ECFCs from diabetic or nondiabetic patients. In this context, Tan et al. [34] injected ECFCs of diabetic and healthy rabbits intramyocardially in diabetic rabbits. They found that injection of transplantation of diabetic ECFCs was not able to rescue the ischemic myocardium of diabetic rabbits. In comparison, transplantation of healthy ECFCs resulted in increased neovascularization and paracrine activation. However, methodological differences might explain the different findings. Tan et al. [34] used recipient animals with severe, untreated diabetes, suggesting that the recipient environment plays an important role for the magnitude of therapeutical effects of cell-based therapies. Likewise, others have reported that the dysfunction of diabetic ECFCs can be restored by improved glycemic control [35,36].

A sole mechanism of action for ECFC beneficial effects remains to be elucidated. In some of these studies, ECFCs facilitate vascular repair by directly integrating within the host vasculature, while in others, vascular engraftment of these cells is low or completely absent [11]. Herein, we could show that ECFC transplantation is associated with a secondary increase in Sca-1^+^ cardiac resident progenitor cells and propose a novel proreparative mechanism of ECFC action. 

In the present study, we were not able to perform noninvasive and repetitive analyses of the animals because mice were sacrificed after invasive hemodynamic measurements and for histological analyses. Therefore, it will be the goal of future studies to use noninvasive imaging techniques such as cardiac MRI to facilitate serial assessments of cardiac function [37].

The rapid donor cell death 30 days after transplantation seen in our study is consistent with previous studies showing poor donor cell survival after transplantation of different cell types [6]. However, reporter gene PET imaging could be employed in future studies to monitor stem cell survival in vivo. Likewise, molecular imaging will possibly help to unravel the mechanistic puzzle about the contributory roles of inflammation, ischemia, apoptosis, anoikis, and autophagy after cell transplantation [38].

## 5. Conclusions

In conclusion, in a murine model of myocardial infarction in SCID mice, transplantation of huECFCs ameliorated myocardial function by attenuation of adverse post-MI remodeling, presumably through paracrine effects. Cardiac repair was enhanced by increasing myocardial neovascularization and the pool of Sca1^+^ cardiac resident stem cells without significant differences in the cardioprotective effects of huECFCs derived from diabetic or nondiabetic patients. The use of huECFCs for treating ischemic heart disease warrants further investigation.

## Supplementary Materials

The following are available online at https://www.mdpi.com/2073-4409/9/3/588/s1, Figure S1: ECFC expression of proangiogenic transcription factors in vitro assessed by RT-PCR.
