# Supplementary material for: Cardioprotective Potential of Human Endothelial-Colony Forming Cells from Diabetic and Nondiabetic Donors
Source: Cells. 2020 Mar 2;9(3):588. doi: 10.3390/cells9030588 (PMC7140510; doi:10.3390/cells9030588)
Supplement: Supplementary file 1 [file cells-09-00588-s001.pdf]

DATA SUPPLEMENT

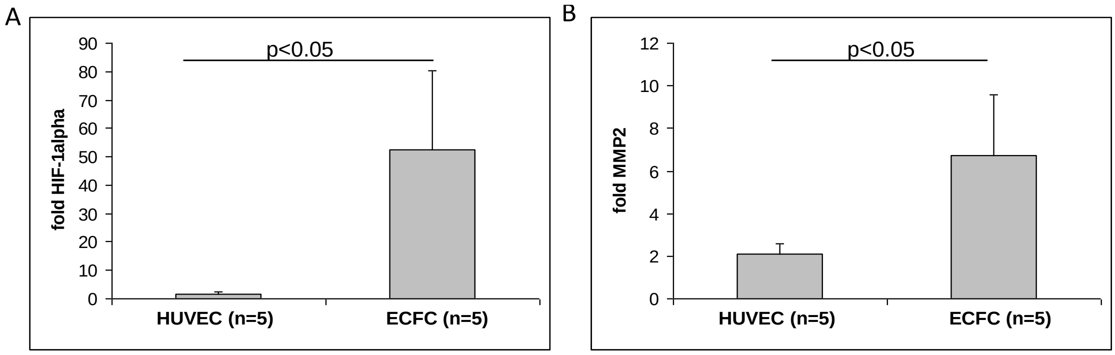

Figure S1. ECFC expression of pro-angiogenic transcription factors *in vitro* assessed by RT-PCR. (A–D) Bar graphs showing increased expression of pro-angiogenic transcription factors HIF-1alpha and MMP-2 in ECFCs compared to HUVEC cells assessed by RT-PCR in vitro (each n=5). Data represent mean ± SEM.

Supplemental Methods

Method real-time PCR (RT-PCR)

huECFCs and HUVEC cells were grown to near confluence as described in the Methods Section. RT-PCR was performed as described previously [13].
